# Detection and Quantification of DNA by Fluorophore-Induced Plasmonic Current: A Novel Sensing Approach

**DOI:** 10.3390/s24247985

**Published:** 2024-12-14

**Authors:** Daniel R. Pierce, Zach Nichols, Clifton Cunningham, Sean Avryl Villaver, Abdullah Bajwah, Samuel Oluwarotimi, Herbert Halaa, Chris D. Geddes

**Affiliations:** Department of Chemistry and Biochemistry, Institute of Fluorescence, University of Maryland, Baltimore County, 701 E Pratt St, Baltimore, MD 21202, USA; dan38@umbc.edu (D.R.P.); z38@umbc.edu (Z.N.); cliftoc1@umbc.edu (C.C.); sean.avryl.14@gmail.com (S.A.V.); abajwah1@umbc.edu (A.B.); soluwar1@umbc.edu (S.O.); hhalaa1@umbc.edu (H.H.)

**Keywords:** plasmonic current, fluorophore induced plasmonic current, plasmonic electricity, metal-enhanced fluorescence, DNA sensing

## Abstract

We report on the detection and quantification of aqueous DNA by a fluorophore-induced plasmonic current (FIPC) sensing method. FIPC is a mechanism described by our group in the literature where a fluorophore in close proximity to a plasmonically active metal nanoparticle film (MNF) is able to couple with it, when in an excited state. This coupling produces enhanced fluorescent intensity from the fluorophore–MNF complex, and if conditions are met, a current is generated in the film that is intrinsically linked to the properties of the fluorophore in the complex. The magnitude of this induced current is related to the spectral properties of the film, the overlap between these film properties and those of the fluorophore, the spacing between the nanoparticles in the film, the excitation wavelength, and the polarization of the excitation source. Recent literature has shown that the FIPC system is ideal for aqueous ion sensing using *turn-on* fluorescent probes, and in this paper, we subsequently examine if it is possible to detect aqueous DNA also via a *turn-on* fluorescent probe, as well as other commercially available DNA detection strategies. We report the effects of DNA concentration, probe concentration, and probe characteristics on the development of an FIPC assay for the detection of non-specific DNA in aqueous solutions.

## 1. Introduction

The concept of fluorophore-induced plasmonic current (FIPC) is at its core based on the foundations of metal-enhanced fluorescence (MEF), also known as plasmon-enhanced or even surface-enhanced fluorescence, which has been extensively reported on in the literature [1,2,3,4,5,6,7,8,9,10,11,12,13,14]. MEF is a phenomenon where, when a fluorophore is in the near field of plasmonically active nanoparticles that share a strong spectral overlap, the two are able to couple, producing a new fluorophore–nanoparticle complex. This excited-state complex retains several of the properties of the fluorophore, such as maximum wavelength of excitation and emission, while differing in others, such as radiative lifetime and quantum yield. The combined complex functions as an antenna, drawing more light into the complex than the fluorophore would have alone, while also altering the electronic nature of the fluorophore, leading to more favorable routes of relaxation and, ultimately, a decreased radiative lifetime. The combination of these two phenomena leads to a substantially increased fluorescence intensity in otherwise identical conditions, compared to the fluorophore alone. This increased fluorescence intensity lends itself to the development of assays and diagnostics, as the increased reporter intensity allows for an enhanced signal dynamic range, while also allowing for a decreased material cost. Recent work by our group has identified that there is a strong relationship between the spacing of the nanoparticles on the film and the intensity of the MEF emission reported [15,16,17,18,19]. It has been found that if the particles become spaced closer to each other in a *semicontinuous* orientation, the MEF signal is reduced in favor of what we describe as a fluorophore-induced plasmonic current (FIPC) across the nanoparticle film [15,16,17,18,19,20,21].

When the coupled complex becomes excited in the FIPC orientation, the nanoparticle itself captures some of this energy and, when it reaches its capacitance limit, releases electrons that are captured by neighboring particles in a process known as electron hopping [15,16]. The charge–discharge cycle produces a detectable current across the surface of the film, and this is further enhanced when a bias current is applied across the film to control the direction of the electron flow, preventing the “canceling out” of electrons going in different directions [15,16]. This induced current has been found thus far to be related to the concentration of fluorophore; the molar extinction coefficient of the fluorophore–nanoparticle complex; the power and polarization of the excitation source; the positioning of the detection electrodes on the surface of the film; the position of the irradiance spot with respect to the electrodes; and the temperature of the system [15,16,17,18,19].

In this paper, we subsequently investigated the FIPC with regard to the analysis of DNA in aqueous solutions, through the means of two different commercially available fluorophores, i.e., ethidium bromide and SYBR Green 1. Ethidium bromide is a staining agent that is well reported in that its fluorescence properties increase ~20× upon its binding with DNA or RNA [20,21,22,23], and recent work has shown that this increase in fluorescent properties can be used as a mechanism for a diagnostic fluorescence assay [20,21,22,23]. SYBR Green 1 is a similar nucleic acid staining agent that displays very similar properties, in that is has very low fluorescence until it intercalates with either DNA or RNA, in which situation its response intensifies greatly and can be used to quantify DNA [24,25,26,27]. Both of these two staining agents are readily available, relatively inexpensive, well reported and studied, and therefore excellent candidates for use in an FIPC assay for the detection of aqueous DNA.

## 2. Materials and Methods

For this study, we used thermally deposited copper nanoparticle films, described and detailed in previous work by our group [18]. In brief, silane-coated glass slides (Sigma, ST. Louis, MO, USA) were cleaned and adhered to the rotary stage of an Edwards auto 306 thermal vapor deposition system, affixed with the silane-coated side closest to the metal sublimation area. The copper metal (Kurt, J, Lesker, Jefferson Hills, PA, USA) was placed into a molybdenum evaporation boat connected in series to a current source. Under a vacuum strength of 2 × 10^−5^ torr, the copper metal was sublimated via current induction heating, and its resulting thickness on the film was determined via an adjacent quartz crystal sensor. The rate of deposition was held constant at 0.1 Å/s throughout the deposition process. The films were then optically and electrically characterized, directly after production, as described in more detail in the literature [18]. These films served as the substrate for our FIPC detection scheme and were used for all successive experiments in this paper.

For the preparation of the fluorescent probe solutions, we followed the manufacturer’s instructions. Ethidium bromide (EtBr) (Invitrogen, Waltham, MA, USA, Lot# 0108002, 10 mg/mL) was supplied by the manufacturer in an aqueous form and was diluted to the desired strength with deionized water. SYBR Green 1 (Sigma, ST. Louis, MO, USA Lot# SLBD5562V, 10,000× in DMSO) solutions were prepared differently compared to how described in the manufacturer’s instructions, in that we did not dilute it using DMSO, as our FIPC system currently utilizes aqueous solutions. The initial 10,000× 1 mL aliquot was thawed directly prior to testing and diluted with DI water to the desired strength. The DNA that we used in this experiment was sheared salmon sperm DNA (Invitrogen, Waltham, MA, USA, Lot# 2701641, 10 mg/mL) and was diluted to the desired strength as well via DI water, based on the manufacturer’s suggestions. The concentrations chosen for DNA and the resulting probes were 1–1000 μg/mL and 1×–100×, in subsequent orders of magnitude, respectively. For the absorption and emission measurements, the solutions were tested directly after reconstitution and mixing using a UV-Vis spectrophotometer (Agilent Technologies, Cary 60 UV-Vis) and a spectrofluorometer (Horiba Fluoromax-4-P). For the emission spectra, both the ethidium bromide–DNA solutions and the SYBR Green 1–DNA solutions were excited at 365 nm.

The FIPC measurements described in this paper were performed using our methodology that has been detailed previously [15,16,17,18,19], with the exact sample methodology shown in [20]. In brief, the copper metal nanoparticle films (MNFs) were directly placed onto the sample stage, as shown in Supplementary Figure S1. Copper electrodes were fixed to the surface and subsequently connected in series to a Keithley 6487 picoammeter. Copper metal was chosen as it was the same metal as that of the nanoparticle film substrate that we were using, and if a silver nanoparticle film were used, silver electrodes would be used. Once the substrate was in place, the desired solution was chosen, and 200 μL of it was deposited onto the film between the two electrodes, while importantly maintaining contact with both of them to reduce resistance in the system. From the far field, a 266 nm solid-state continuous-wavelength (CW) laser was warmed for 15 min prior to testing and was aligned such that when the gating was open, it would strike the surface of the film at an incidence angle of 45°, as also outlined in [18]. The purpose of this geometry was to allow for the selective excitation of those fluorophores in phase, rather than of those out of phase, with the dipoles of the nanoparticle films, through the use of **S-** and **P**-polarized excitation light, determined using a half-wave plate [20]. From the FIPC data obtained for EtBr with and without DNA, we determined that at concentrations between 40 μM and 60 μM, there was the largest current range between the two, and as such, 50 μM was chosen as the concentration of choice for the EtBr experiments. Considering this information, DNA concentrations of 1 μg/mL–10 mg/mL were chosen to be tested using the selected EtBr concentration. Absorption and emission data were collected for all of the combinations tested and are detailed in the following results section. The initial concentration determination for SYBR Green 1 was expedited after the initial findings from the EtBr determinations, and the concentrations of 1×, 10×, and 100× were chosen (please note that as SYBR Green 1 is a proprietary probe, no concentration information other than “initial concentration 10,000×” was provided). While we expected that concentrations higher than 2× would prove to be less reliable and exceed the limits of the spectrophotometers, we were curious to see their impact on the FIPC setup, which has shown in the past not to have the same dynamic range limitations that traditional fluorescence sensing has. For these probe concentrations, 1 μg/mL–1 mg/mL of DNA was used. The FIPC data were collected for these samples identically as for the EtBr samples, and the corresponding spectra can be found in the Results section below.

## 3. Results and Discussion

### 3.1. Detection of Aqueous DNA Using Ethidium Bromide

**Figure 1:**
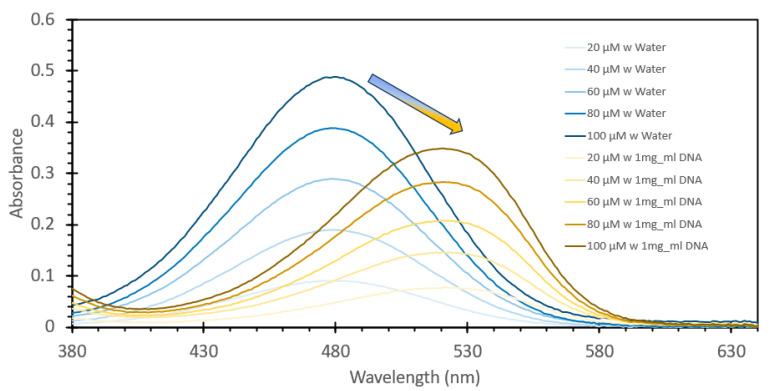
Absorption spectra of various solutions of ethidium bromide, ranging from 20 µM to 100 µM, both with and without the addition of 1 mg/mL DNA from a salmon sperm DNA stock solution. The solutions were allowed to mix for 30 min prior to analysis.

**Figure 2:**
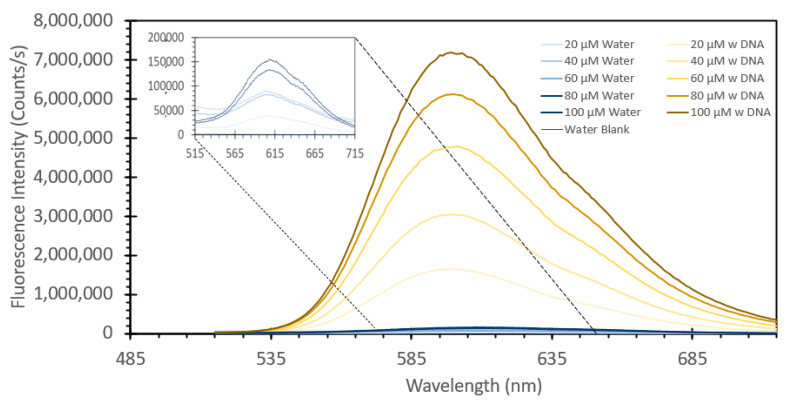
Fluorescence emission spectra of various solutions of ethidium bromide, ranging from 20 µM to 100 µM, both with and without the addition of 1 mg/mL DNA from a salmon sperm DNA stock solution, excited at 365 nm. The solutions were allowed to mix for 30 min prior to analysis.

**Figure 3:**
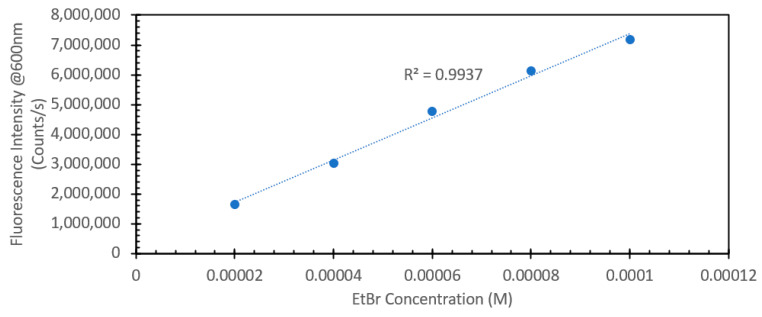
Peak fluorescence emission spectra values for various solutions of ethidium bromide, ranging from 20 µM to 100 µM with the addition of 1 mg/mL DNA from a salmon sperm DNA stock solution, excited at 365 nm. The solutions were allowed to mix for 30 min prior to analysis. Data organized as the peak fluorescence intensity at 600 nm vs. concentration.

**Figure 4:**
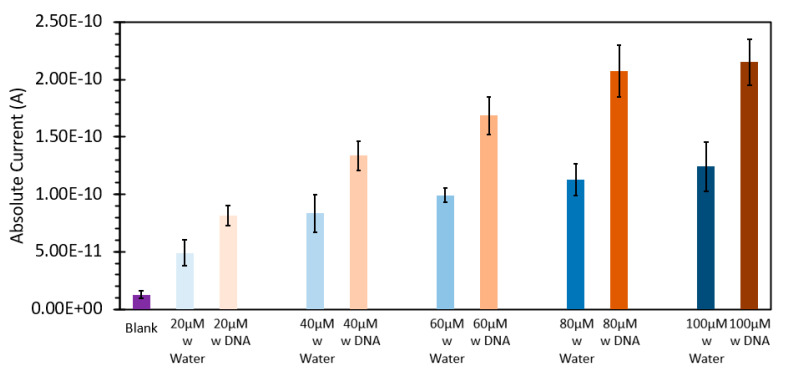
Plasmonic current response of various solutions of ethidium bromide, ranging from 20 µM to 100 µM, both with and without the addition of 1 mg/mL DNA from a salmon sperm DNA stock solution, excited at 266 nm at 100 µW excitation power. The solutions were allowed to mix for 30 min prior to analysis.

**Figure 5:**
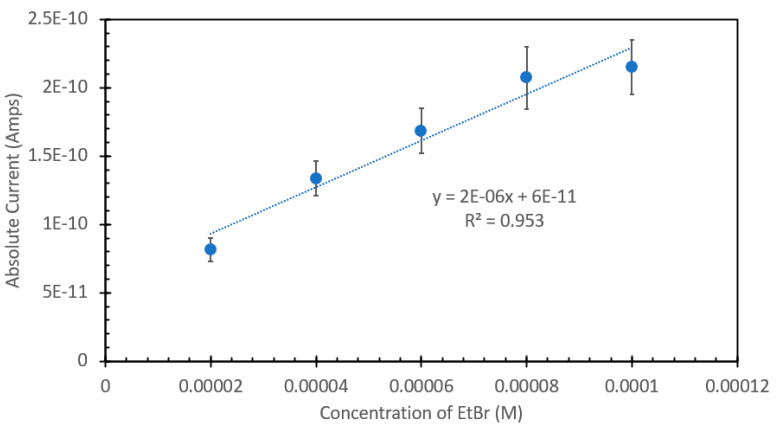
Plasmonic current response of various solutions of ethidium bromide, ranging from 20 µM to 100 µM with the addition of 1 mg/mL DNA from a salmon sperm DNA stock solution, excited at 266 nm at 100 µW excitation power. The solutions were allowed to mix for 30 min prior to analysis.

Ethidium bromide is a common staining agent widely used for the visualization of DNA in agarose gels and has been shown in the literature to have the capacity to be used as a method of DNA quantification in aqueous samples [20,21,22,23]. To confirm what was noted in the literature, we decided to test the responsive characteristics of EtBr to both changes in concentration of itself relative to a fixed amount of DNA, and once a suitable concentration was found, for maximum FIPC measurement, subsequently testing it against a wide range of DNA concentrations. Figure 1 and Figure 2 show the initial results using a fixed solution of salmon sperm DNA (1 mg/mL) treated with varying concentrations of EtBr, starting from 20 μM and increasing to 100 μM. Figure 1 shows the absorbance spectra of the solutions, with a marked shift of one of the well-defined absorbance peaks at ~480 nm for the samples without DNA to ~525 nm for those with DNA. It was also noted that there was a marked and incremental increase in intensity for both peaks as the concentration of EtBr increased. Figure 2 shows the fluorescence spectra of these solutions excited at 266 nm (excitation wavelength of choice for the subsequent FIPC tests), with the solution with water substituted for DNA being shown in the figure inset. Peak emission was noted at ~600 nm, with an incremental intensity increase based on the increasing concentration of EtBr, whereas little response was noted for the solutions of EtBr without DNA. Figure 3 is a representation of the linear peak fluorescence responses for the solutions of EtBr with DNA addition, compared to what we noted in the literature [20,21,22,23], and it was noted that there was a strong relationship between peak fluorescence intensity and EtBr concentration, exemplified by the R^2^ of the trendline being >0.99. Figure 4 shows the plasmonic current responses for the five examined concentrations, under excitation at 266 nm at 100 μW power, both with and without DNA. From the data, we were interested in finding the largest difference between the EtBr solutions both with and without DNA, as this would help determine which had the highest FIPC response relative to the overall generated plasmonic current. It was subsequently determined that at concentrations between 40 μM and 60 μM, we recorded the highest responses relative to the water control and their non-DNA doped counterparts. Subsequently 50 μM EtBr was chosen for the future experiments. Figure 5 shows the peak FIPC values for the five examined solutions of EtBr that contained DNA, with an average of 30 values for each data point. Figure 5 shows that the FIPC responses had modest linearity over the same range, with an R^2^ value of ~0.95, similar to what observed for the fluorescence responses alone (Figure 3 vs. Figure 5).

**Figure 6:**
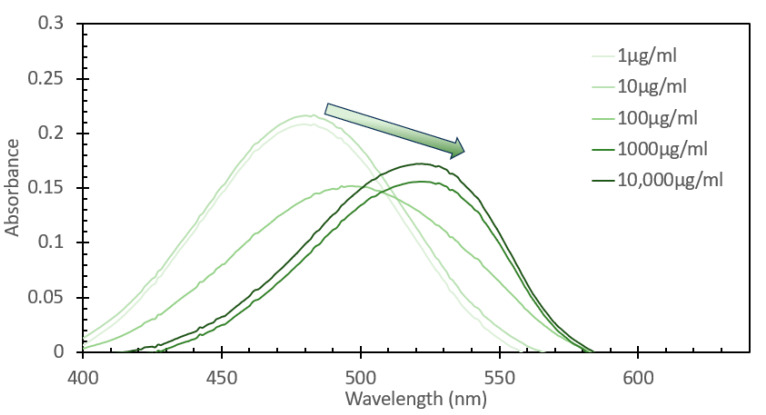
Absorbance spectra of a 50µM solution of ethidium bromide mixed with varying concentrations of DNA from a salmon sperm DNA stock solution, ranging from 1 µg/mL to 10,000 µg/mL. The solutions were allowed to mix for 30 min prior to analysis.

**Figure 7:**
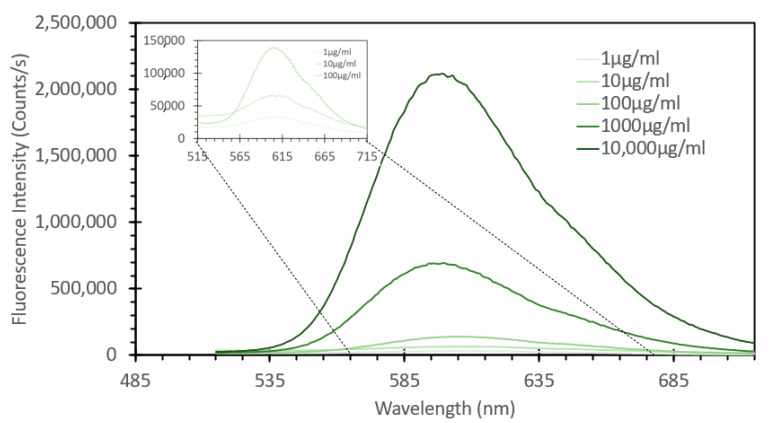
Fluorescence emission spectra of a 50 µM of ethidium bromide mixed with varying concentrations of DNA from a salmon sperm DNA stock solution, ranging from 1 µg/mL to 10,000 µg/mL, excited at 266 nm. The solutions were allowed to mix for 30 min prior to analysis.

**Figure 8:**
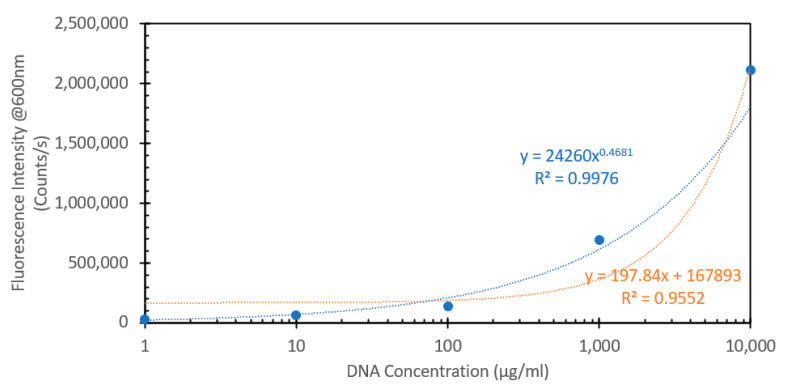
Peak fluorescence emission spectra values of a 50 µM of ethidium bromide solution mixed with varying concentrations of DNA from a salmon sperm DNA stock solution, ranging from 1 µg/mL to 10,000 µg/mL, excited at 266 nm. The solutions were allowed to mix for 30 min prior to analysis. Data shown as peak fluorescence intensity at 600 nm vs. DNA concentration.

**Figure 9:**
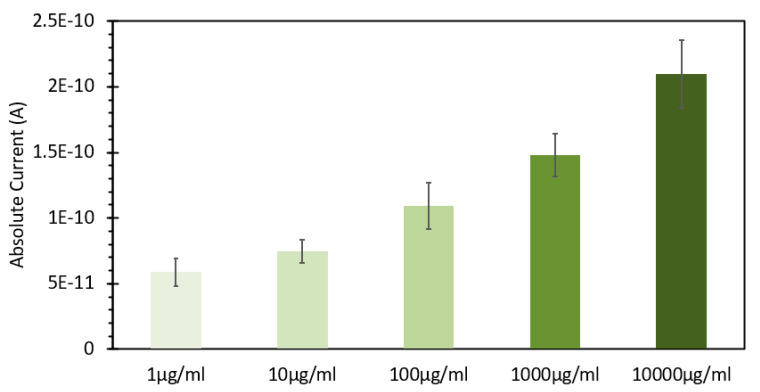
Plasmonic current response of a 50 µM of ethidium bromide solution mixed with varying concentrations of DNA from a salmon sperm DNA stock solution, ranging from 1 µg/mL to 10,000 µg/mL, excited at 266 nm at 100 µW power. The solutions were allowed to mix for 30 min prior to analysis.

From the data shown in Figure 4, a subsequent experiment was designed to determine the range over which this concentration of probe would be able to detect DNA. Fixed solutions of 50 μM EtBr were subsequently spiked with increasing amounts of salmon sperm DNA, ranging from 1 μg/mL to 10,000 μg/mL. Figure 6 shows the absorbance spectra of these solutions, and what can be noted is that similar to the absorbance spectra of the solutions with and without DNA shown in Figure 1, we see a red shifting of the peak absorbance upon increasing the DNA concentration, but only to a point, as above 1000 μg/mL, we see no further shifting, but rather just an increase in peak absorption. Figure 7 shows the fluorescence spectra of these solutions excited at 266 nm, consistent with both Figure 2 and the FIPC experiments using the 266 nm solid-state laser. It can be seen that the fluorescence intensity dramatically increased as the concentration of DNA increased, as the lowest value was ~25,000 counts/s, and the highest one was close to 2,500,000 counts/s, a 100× increase over a 10,000× change in the DNA concentration. It was apparent that, over this range, we did not see evidence of a well-established linear fit, as displayed in Figure 8, which shows the linear fit of peak fluorescence at 600 nm (in orange) and the associated R^2^ that was only ~0.95. Also, in Figure 8, shown in blue, is a power-based fit, which had a better R^2^, indicating a slightly better fit. Supplemental Figure S2 details the same data as Figure 8 but with the 10,000 μg/mL data point removed, and we now see a marked return to linearity, with R^2^~0.997; for subsequent experimentation, the 10,000 μg/mL DNA concentration was no longer considered. Figure 9 shows the FIPC responses of these solutions, and we see that there was an increasing response relative to increasing DNA concentrations. We also noted that there was not a dramatic increase in the current response relative to the order of magnitude of the changes in the DNA concentration, particularly for high concentrations. Subsequently, compared to traditional fluorescence, we concluded that for our FIPC approach, using EtBr would not be the appropriate combination; so, we turned our attention to another commercially available DNA probe, namely, SYBR Green 1.

### 3.2. Detection of Aqueous DNA Using SYBR Green 1

**Figure 10:**
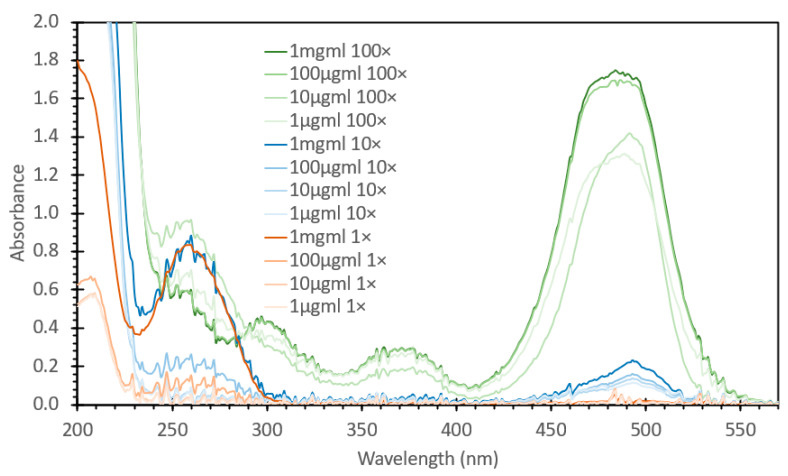
Absorbance spectra of various solutions of SYBR Green with varying concentrations of added DNA. Concentrations in μg/mL and mg/mL are related to DNA concentration added; 100×, 10× and 1× are related to the concentrations of SYBR Green.

**Figure 11:**
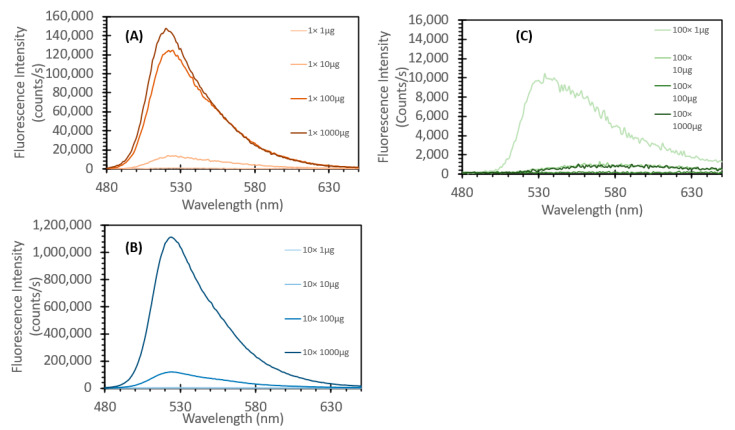
Fluorescence spectra of various solutions of SYBR Green with different concentrations of DNA added, excited at 473 nm. (**A**) 1× Concentration, (**B**) 10× Concentration and, (**C**) 100× Concentration of SYBR Green.

**Figure 12:**
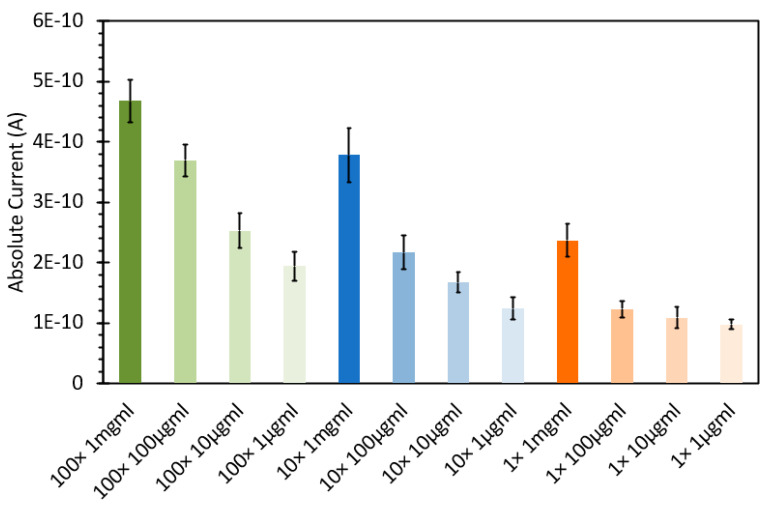
FIPC responses for various solutions of SYBR Green with various amounts of DNA added, excited via a 473 nm (CW) laser, 10 mW power.

SYBR Green 1 is a DNA-intercalating probe that is widely used for visualizing DNA, typically during agarose gel separation. Recent research has shown that this fluorescent stain can also be used for DNA quantification via aqueous fluorescence assays [24,25,26,27]. Similarly to what we had previously examined with the initial ethidium bromide testing, we initially set about to determine if SYBR Green 1 would be useful to detect DNA in an FIPC configuration. We reconstituted the frozen 10,000× SYBR Green as per the manufacturer’s instructions but using a slightly different solution, namely, a 50/50 (*v/v*) ethanol/ water solution. As the molecular weight and structure of SYBR Green 1 are proprietary, the concentrations are here listed in various forms of “X” based on the initial 10,000× concentration of the stock solution, with 1× being the manufacturer’s recommended concentration for use in staining an agarose gel for DNA detection. We prepared 100×, 10× and 1× solutions and then treated them with salmon sperm DNA at concentrations ranging from 1 μg/mL to 1000 μg/mL, increasing by orders of magnitude. Based on the previous experimental findings with EtBr, we omitted the 10,000 μg/mL concentration from the testing. The absorbance spectra of these 12 solutions are shown in Figure 10, and what can be noted is that the peak absorbance was observed around ~470 nm, with increasing absorbance at this wavelength based on both the concentration of SYBR Green and that of the DNA added. Based on the data shown in Figure 10, we noted a large difference between the SYBR Green concentrations, with each concentration also showing an increase in absorption with an increasing DNA concentration. Figure 11 shows the subsequent fluorescence spectra of these solutions, excited at 473 nm, in line with the FIPC testing at the same wavelength. For the 1× concentration, we saw a systematic increase in fluorescence intensity with an increasing concentration, with a small difference with solutions at concentrations between 100 μg/mL and 1000 μg/mL relative to the change observed with solutions at concentrations between 10 μg/mL and 100 μg/mL. For the 10× concentration, we saw that the fluorescence intensity with the 100 μg/mL and 1 mg/mL solutions was substantially higher than that with the others, and that there was no evidence of saturation of the probe relative to the DNA concentration. For the 100× concentration, we saw that there was evidence of the inner filter effect, as the solution had barely any fluorescence response. Figure 12 shows the FIPC responses of the 12 solutions, excited at 473 nm and 10 mW power, with each error bar calculated from 30 data points. We readily see that there is a relationship between increased probe concentration and FIPC response, as well as between FIPC response and increased DNA concentration for each probe concentration range.

**Figure 13:**
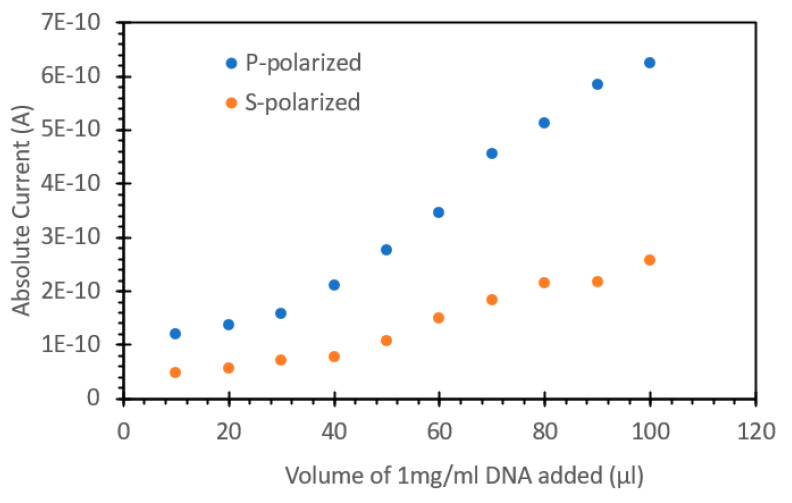
FIPC response for 1× SYBR Green with added DNA, excited via 473 nm laser. Comparison between P and S polarized light for excitation.

**Figure 14:**
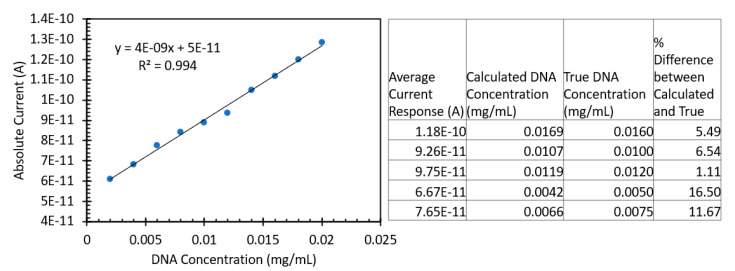
Standard curve obtained using 1× SYBR Green and various DNA concentrations (Left). In a blinded study, the sample values were found to be within 15% of their true value. Table detailing the raw responses and their calculated values (on the right). Each current response shown is the average of 10 values, n = 10.

Finally, we tested the influence of excitation polarization upon DNA detection. Figure 13 shows the effects of **S** and **P** excitation polarization. Over the range chosen, we observed a trend of increasing **P** polarization-induced current at a higher rate than the **S** polarization-induced current responses, indicating that this would be a suitable range for the development of an FIPC assay using SYBR Green. Figure 14 shows a standard curve developed using the DNA concentrations of 0.002 and 0.02 mg/mL and the averages of 30 FIPC values. Five blinded samples were subsequently tested, and we achieved results within ~15% of the true values of the testing solution. These blinded samples were prepared by a researcher not directly involved in the same testing via FIPC and involved the dilution of the stock salmon sperm DNA to concentrations into the range of those used for the standard curve shown in Figure 14. Five samples were prepared, whose concentrations were written down, but not shared with the FIPC analyst. After calculating the determined concentrations from the standard curve, the two concentration values were compared to each other.

## 4. Conclusions

In this report, we examined the effects of using commercially available fluorescent DNA stains as a method for the detection of aqueous DNA, using fluorophore-induced plasmonic current. We demonstrated that these stains can be used as probes for both fluorescence and FIPC measurements due to their increasing fluorescence intensity in response to increased aqueous DNA concentration. We found that, while ethidium bromide demonstrated increased plasmonic current responses, its sensitivity was low relative to that observed for SYBR Green, and the latter was subsequently tested via a blinded study to determine its efficacy. The ability to adapt FIPC into a platform for the detection of DNA provides researchers and analysts alike with a new method for the immediate detection of DNA in solutions, directly at the point of sample collection, potentially decreasing the effects of sample degradation prior to analysis. Interestingly, while both EtBr and SYBR Green 1 are fluorescent DNA-intercalating stains/probes, no fluorescence emission was actually measured directly using our FIPC methodology. Subsequently, we envision that the many fluorescent assays employed today can be simply switched over to an FIPC methodology, significantly reducing the instrumentation costs for sensing, i.e., dispensing from the use of a fluorometer. Further details will be reported by our group in due course.

## Data Availability

Data are contained within the article.

## Supplementary Materials

The following supporting information can be downloaded at: https://www.mdpi.com/article/10.3390/s24247985/s1. Supplemental Figure S1: Experimental collection of plasmonic current. Excitation light from a CW laser is passed through a neutral density filter before falling incident on the sample stage, exciting the fluorophore on the surface of the film. Fluorophore-induced plasmonic currents are collected through electrodes in series with a picoameter, and fluorescence emission is collected via a fiber optic cable. Supplemental Figure S2: Peak fluorescence emission spectra values of a 50 μM of ethidium bromide solution mixed with varying concentrations of DNA from a salmon sperm DNA stock solution, ranging from 1 μg/mL to 1000 μg/mL, excited at 266 nm. The solutions were allowed to mix for 30 min prior to analysis. Data organized as peak fluorescence intensity at 600 nm vs. DNA concentration
