# Supplementary material for: Detection and Quantification of DNA by Fluorophore-Induced Plasmonic Current: A Novel Sensing Approach
Source: Sensors (Basel). 2024 Dec 14;24(24):7985. doi: 10.3390/s24247985 (PMC11679120; doi:10.3390/s24247985)
Supplement: Supplementary file 1 [file sensors-24-07985-s001.zip › sensors-3356765-supplementary.pdf]

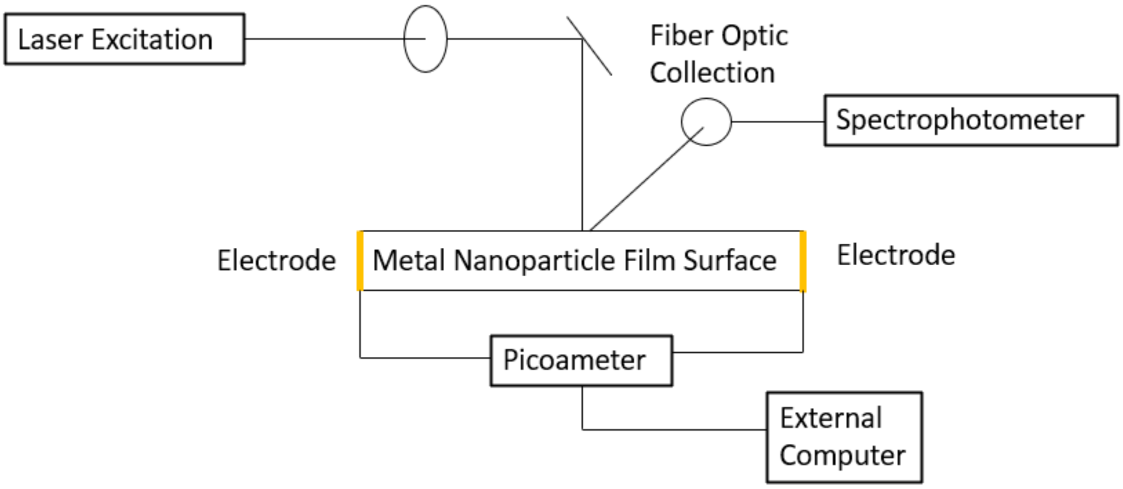

Supplemental Figure 1 – Experimental collection of plasmonic current. Excitation light from a CW laser is passed through a neutral density filter before falling incident on the sample stage, exciting the fluorophore on the surface of the film. Fluorophore-induced plasmonic currents are collected through electrodes in series with a picoameter, and fluorescence emission is collected via a fiber optic cable.

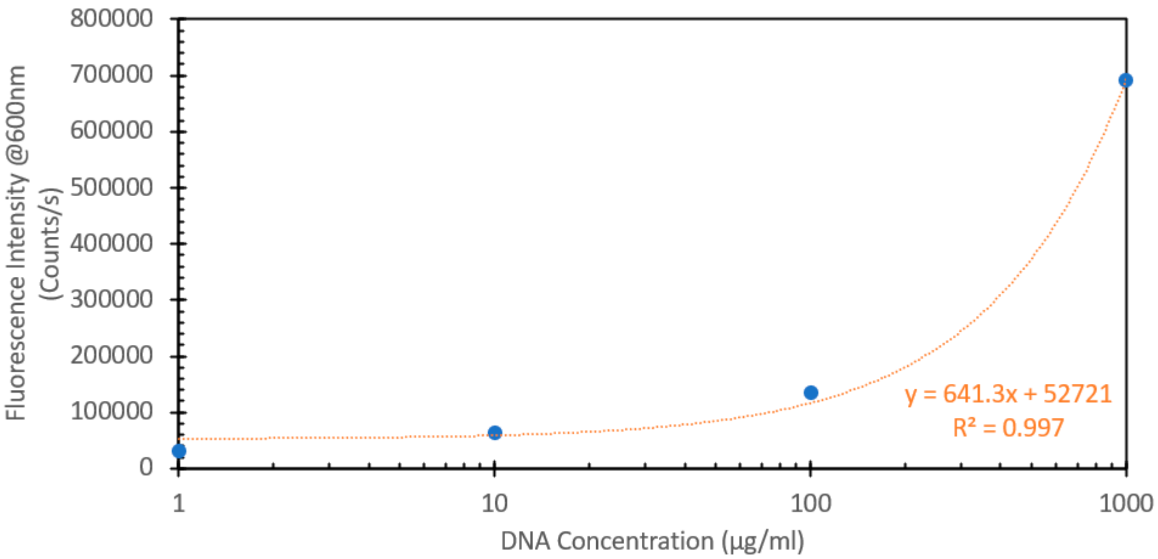

Supplemental Figure 2- Peak fluorescence emission spectra values of a 50µM of ethidium bromide solution mixed with varying concentrations of DNA from a salmon sperm DNA stock solution, ranging from 1µg/ml to 1000µg/ml, excited at 266nm. The solutions were allowed to mix for 30 minutes prior to analysis. Data organized as peak fluorescence intensity at 600nm vs DNA concentration.

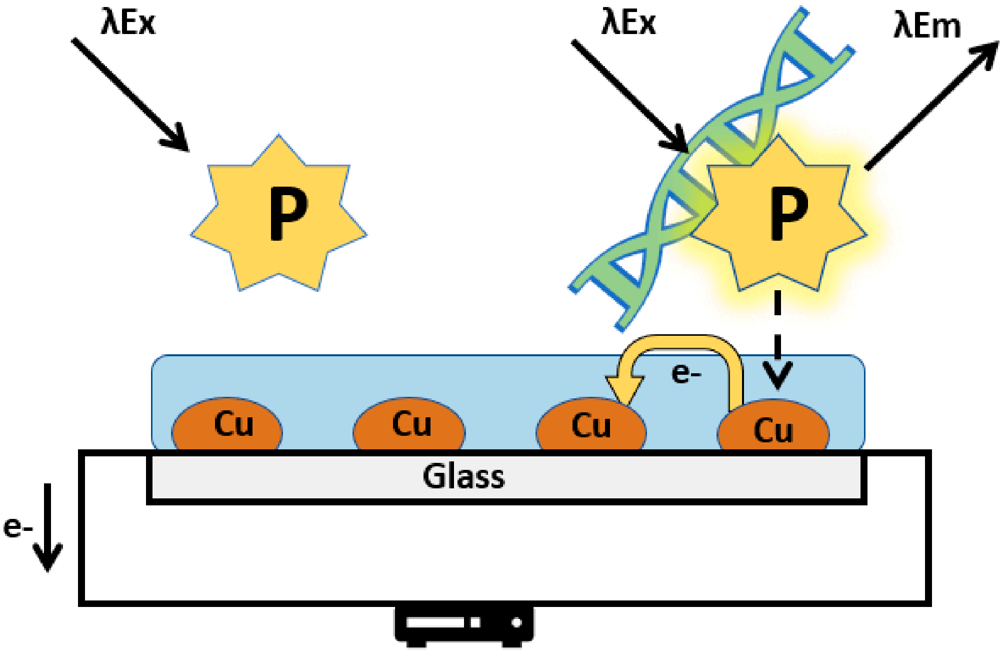

TOC GRAPHIC – Ball and stick diagram detailing the *turn on* fluorescence of the various DNA detection probes used in this study. When fluorescence is present the energy transfer to produce fluorophore-induced plasmonic current occurs.
